# Cytotoxic Activity of the Red Grape Polyphenol Resveratrol against Human Prostate Cancer Cells: A Molecular Mechanism Mediated by Mobilization of Nuclear Copper and Generation of Reactive Oxygen Species

**DOI:** 10.3390/life14050611

**Published:** 2024-05-09

**Authors:** Mohd Farhan

**Affiliations:** 1 Department of Chemistry, College of Science, King Faisal University, Al Ahsa 31982, Saudi Arabia; mfarhan@kfu.edu.sa; 2 Department of Basic Sciences, Preparatory Year, King Faisal University, Al Ahsa 31982, Saudi Arabia

**Keywords:** resveratrol, prostate cancer cells, reactive oxygen species, pro-oxidant, apoptosis

## Abstract

Resveratrol, a polyphenolic compound found primarily in red grapes and pomegranates is known as an antioxidant but can act as a pro-oxidant when copper ions are present. Here, resveratrol is demonstrated to reduce cell growth (as evaluated by MTT assay) and promote apoptosis-like cell death (as measured by Histone/DNA ELISA) in prostate cancer cell lines PC3 and C42B. This effect is effectively inhibited by a copper chelator (neocuproine) and reactive oxygen species (ROS) scavengers (thiourea for hydroxyl radical, superoxide dismutase for superoxide anion, and catalase for hydrogen peroxide). These inhibitory effects provide evidence that intracellular copper reacts with resveratrol within cancer cells, resulting in DNA damage via the generation of reactive oxygen species. Additionally, it has been demonstrated that non-tumorigenic epithelial cell lines (MCF-10A) grown in media supplemented with copper are more susceptible to growth inhibition by resveratrol, as confirmed by the observed reduction in cell proliferation. Copper supplementation induces enhanced expression of the copper transporter *CTR1* in MCF-10A cells, which is reduced by the addition of resveratrol to the media. The selective cell death of cancer cells generated by copper-mediated and ROS mechanisms may help to explain the anticancer properties of resveratrol.

## 1. Introduction

Resveratrol, also known as *trans*-3,5,4′-trihydroxystilbene, is a stilbenoid. The amount of resveratrol found in red grape skin is the highest. Tea, blueberries, pomegranates, almonds, pistachios, and dark chocolate are among the foods that have also been found to contain resveratrol [1]. Research on resveratrol has recently picked up interest due to the so-called “French Paradox”. This refers to the seemingly contradictory observation that people in Southern France have a relatively low rate of coronary heart disease despite drastically high saturated fat consumption. Studies have linked this incidence to a higher intake of red wine in this area, notably resveratrol, which is the key element responsible for this action [2,3].

In addition to its well-established advantages in preventing cardiovascular diseases, resveratrol has several other reported effects, including anti-inflammatory, anti-platelet, hyperlipidemic, immunomodulatory, anti-carcinogenic, cardioprotective, and neuroprotective activity [4,5,6,7,8,9,10]. Resveratrol inhibits LDL oxidation [11], for which it has been proposed to chelate copper ions [12]. Resveratrol possesses a very high antioxidant potential and has been suggested as a potential cancer chemopreventive drug that can halt the development of tumors at any point in the carcinogenesis process, including the initiation, promotion, and progression stages [1,13]. Such preventative anticancer action in colon, cervical, prostate, breast, and lung cancers has been shown in several *in vitro* and animal-based investigations worldwide [14,15,16,17,18,19,20]. In recent times, researchers have also performed multiple human clinical trials to validate the anticancer properties of resveratrol, aiming to obtain a deeper understanding of its potential clinical significance [21,22,23,24,25].

The author’s lab and his parent lab have previously shown that curcumins [26], genistein [27], flavonoids [28], catechins [29], and gallic acid [30] are plant-based antioxidant polyphenolic compounds that can cause oxidative DNA damage when combined with specific transition metal ions, particularly copper “Cu(II)”. Resveratrol, a polyphenol, can also cause DNA strand breaks when exposed to Cu(II) [31]. Using a cellular system of lymphocytes isolated from human peripheral blood and alkaline single-cell gel electrophoresis (comet assay), it has already been confirmed that the resveratrol–Cu(II) system is indeed capable of causing DNA degradation in cells such as lymphocytes [32].

Copper is a major metal ion present in chromatin and is tightly linked to DNA bases, especially guanine [28,33], and it is a highly redox-active metal ion present in living cells. Cancer patients exhibit considerably increased levels of copper in their blood, tissues, and cells, as indicated by many studies [33,34]. This is a very compelling discovery, and, interestingly, the increase in copper in the tumor is not determined by the tissue type but rather reflects a metabolic characteristic of the tumor [35]. However, it is unclear if the systemic rise in copper is a cause or a result of the malignant transformation. Most plant polyphenols (including resveratrol) exhibit both antioxidant and pro-oxidant characteristics [1,6,33,36,37]. Previous studies have suggested that the pro-oxidant behavior of polyphenolics could play a crucial role in their ability to show an anticancer effect and induce apoptosis [26,27,32,33,38]. The mobilization of endogenous copper ions may provide a pro-oxidant effect that could be a potential mechanism for how these polyphenols selectively eliminate cancer cells.

**Figure 1:**
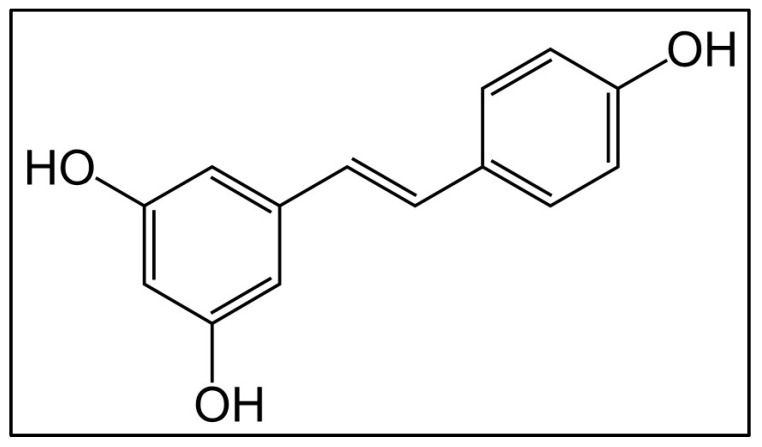
The chemical structure of resveratrol.

In the article, the author verifies the mechanism of action of polyphenolic compounds derived from plants through the utilization of resveratrol. In prostate cancer cell lines, resveratrol has been shown to inhibit cell proliferation and promote apoptosis. Neocuproine, a cuprous chelator, and ROS scavengers such as catalase, superoxide dismutase, and thiourea notably impede this type of cell death. Copper chelation inhibits this ROS formation, validating the conclusion that the mobilization of intracellular copper by resveratrol results in the development of ROS that induces pro-oxidant cell death. Furthermore, resveratrol-induced growth inhibition effects are observed in MCF-10A cells (non-tumorigenic epithelial cells) cultured in a medium containing copper. The significance of the copper transporter gene *CTR1* in the survival dynamics of malignant cells subsequent to resveratrol exposure is also illustrated by the author. The structure of resveratrol is shown in Figure 1.

## 2. Materials and Methods

### 2.1. Cell Lines and Reagents

The non-tumorigenic breast epithelial cell line MCF-10A (CRL-10317) and prostate cancer cell lines PC3 (CRL-1435) and C42B (CRL-3315) were acquired from ATCC in Manassas, VA, USA. PC3 and C42B cells were cultured in RPMI medium from Invitrogen (Carlsbad, CA, USA). The media contained 10% fetal bovine serum, 100 units/mL penicillin, and 100 µg/mL streptomycin. The cells were grown in a 5% CO_2_-humidified environment at 37 °C. Resveratrol stock solutions at a concentration of 50 mM were prepared in dimethyl sulfoxide (DMSO) and stored in tiny aliquots at −20 °C. Stock solutions of several metal ion chelators such as neocuproine, desferoxamine mesylate, and histidine were prepared in phosphate buffered saline (PBS) at a final concentration of 50 mM. These solutions were always freshly prepared right before experiments. The specific concentration of resveratrol and metal chelators utilized is indicated in each individual experiment.

### 2.2. Cell Growth Inhibition Investigations by 3-(4,5-Dimethylthiazol-2-yl)-2,5-diphenyltetrazolium Bromide (MTT) Assay

The MTT assay is a colorimetric method designed to screen cell viability. This assay quantifies the decrease in yellow MTT (3-(4,5-dimethylthiazol-2-yl)-2,5-diphenyltetrazolium bromide) to a solid blue formazan product by the enzyme mitochondrial succinate dehydrogenase. Succinate dehydrogenase is an enzyme that plays a role in both the tricarboxylic acid cycle (also known as the Krebs cycle) and complex II of the mitochondrial respiratory chain. The MTT assay was conducted following the previously provided instructions [26]. Cells were seeded at a density of 2 × 10^3^ cells per well on 96-well microtiter culture plates. Following an overnight incubation, cells were subjected to different treatments and/or various chelators, as specified in individual experiments. The absorbance was recorded at a wavelength of 595 nm using the Ultra Multifunctional Microplate Reader from TECAN in Durham, NC, USA. Each treatment consisted of eight duplicate wells, and the concentration of DMSO in the reaction mixture did not surpass 0.1%. Furthermore, each experiment was conducted a minimum of three times. Cell proliferation inhibition in each treatment is expressed as a percentage of the treated cell number relative to the untreated control cells.

### 2.3. Apoptosis Detection Using A Histone/DNA ELISA

The Cell Death Detection ELISA Kit from Roche in Palo Alto, CA, USA was utilized to identify apoptosis in cancer cells exposed to resveratrol, following the manufacturer’s instructions as described previously [26]. Chelators, scavengers, or controls were added separately in each experiment as specified in individual experiments. The samples’ spectrophotometric absorbance was measured at 405 nm using the Ultra Multifunctional Microplate Reader (TECAN).

### 2.4. Soft Agar Colonization Assay

In 24-well plates, 3 × 10^4^ cancer cells were seeded in 0.5 mL of culture medium with 0.3% *w*/*v* top agar on top of a 0.7% *w*/*v* basal layer of agar (together with the culture medium and supplements). Resveratrol or DMSO was added to the culture during seeding, with or without neocuproine as specified in experiment. Colony counts were taken after the recommended 22-day culture period. The experiments were repeated three times, and the average results were presented.

### 2.5. Culture of Normal Epithelial Cells with Copper Enrichment

The MCF-10A, non-tumorigenic, epithelial cell line was cultured in DMEM/F12 (Invitrogen) with the following components added: 5% horse serum, 20 ng/mL EGF, 0.5 µg/mL hydrocortisone, 0.1 µg/mL cholera toxin, 10 µg/mL insulin, 100 units/mL penicillin, and 100 µg/mL streptomycin. The culture was maintained at 37 °C in a 5% CO_2_ environment. The MCF-10A cells cultivated with 25 µM CuCl_2_ for at least 4 weeks in addition to their usual culture media are referred to as MCF-10A+Cu cells, following the protocol as reported earlier [26].

### 2.6. Migration of Cells Assay

Transwell permeable supports with 24 wells and 8 mm holes from Corning, New York, USA, were used to conduct a cell migration assay. The transwell embeds were seeded with cells that had been suspended in a serum-free media. The complete media was inserted into the bottom wells. Next, the cells were fixed with 4 mg/mL calcein AM (Invitrogen, Carlsbad, CA, USA) in PBS for 1 h at 37 °C after 24 h. Then, they were trypsinized to remove them from the inserts. A TECAN Ultra Multifunctional Microplate Reader (Durham, NC, USA) was used to read the migrating cells’ fluorescence.

### 2.7. Reverse Transcriptase PCR in Real-Time

Total RNA was extracted using TRIzol (Invitrogen) reagent following the manufacturer’s instructions. mRNA levels were measured using real-time PCR. The primers used for *CTR1* and *GADPH* (Table 1) were identical to those previously described [26]. RNA levels were standardized based on *GAPDH* expression.

### 2.8. Transfection of Small Interfering RNAs (siRNAs)

The siRNA transfections were performed as previously described [26]. Santa Cruz Biotechnology, Inc. was approached regarding *CTR1*-specific siRNA. A garbled siRNA was used as the control. Transfections were performed using Lipofectamine RNA iMAX Transfection Reagent (Invitrogen) according to the manufacturer’s instructions. *CTR1* was silenced using siRNA 48 h before the experiment.

### 2.9. Statistical Data Analysis

The data for the statistical analysis, which is the standard error of the mean (±S.E.) for three separate experiments, were obtained using the procedures described by Tice et al. [39]. For the purpose of analyzing statistical significance, a Student’s *t*-test was employed. Analysis of variance was carried out using ANOVA. A *p*-value less than 0.05 was considered statistically significant.

## 3. Results

### 3.1. Resveratrol Suppresses Proliferation and Induces Apoptosis in Prostate Cancer Cells

**Figure 2:**
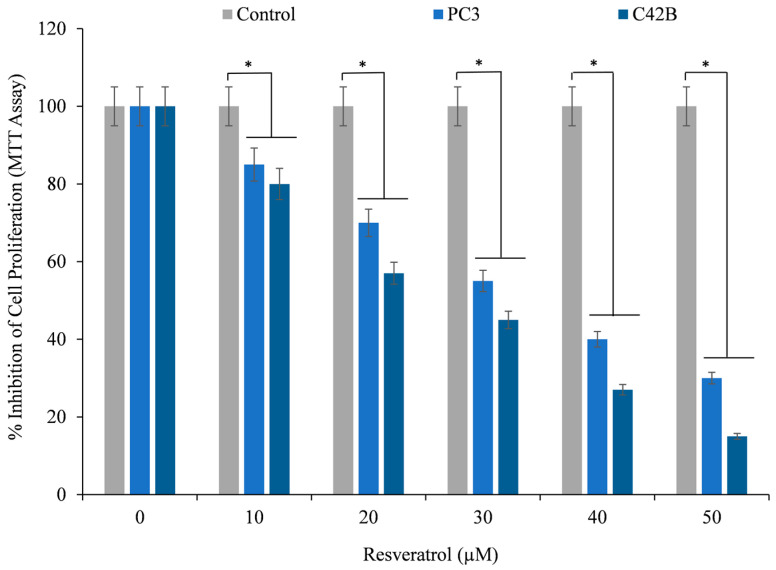
The prostate cancer cell lines, PC3 and C42B, were cultured with specified doses of resveratrol for 72 h. The effect on cell proliferation was determined using the MTT test, as outlined in Section 2. The data are presented as a percentage of the control value, with the standard error (±S.E.) calculated from three independent experiments. * *p* < 0.05 indicates a statistically significant difference compared to the corresponding untreated control (i.e., 0 µM resveratrol).

An investigation into the effects of resveratrol on the inhibition of cancer cell proliferation was carried out with the MTT assay. After being subjected to varying concentrations of resveratrol, cells derived from the prostate cancer cell lines PC3 and C42B were examined. Figure 2 demonstrates that the growth inhibition of both types of prostate cancer cells was dependent on the resveratrol concentration. Furthermore, resveratrol was found to have a more potent inhibitory impact on C42B cells in comparison to PC3 cells, as demonstrated by the findings.

**Figure 3:**
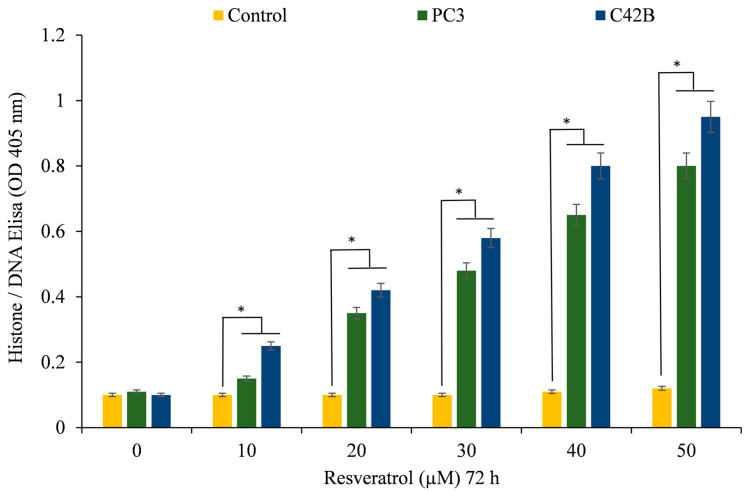
The Histone/DNA ELISA technique was employed to identify apoptosis in cells from the PC3 and C42B prostate cancer cell lines. The cells were incubated for 72 h with varying doses of resveratrol, as shown in the figure and outlined in Section 2. The values presented represent the standard error (±S.E.) of three separate studies. * *p* value < 0.01, compared to control.

In addition, Histone/DNA ELISA was utilized to evaluate the apoptotic induction of resveratrol in the two prostate cancer cell lines that were mentioned earlier (Figure 3). According to these findings, resveratrol was able to suppress the proliferation of cancer cell lines and trigger apoptosis in those cells. Previous studies have demonstrated that resveratrol possesses anticancer activities against a variety of cancer cell lines [1,15,17,18,19,21,40]. These findings provide further evidence that resveratrol possesses anticancer properties.

### 3.2. Cuprous Chelator Inhibits Resveratrol-Induced Antiproliferation and Apoptosis in Cancer Cells but Not Iron and Zinc Chelators

**Figure 4:**
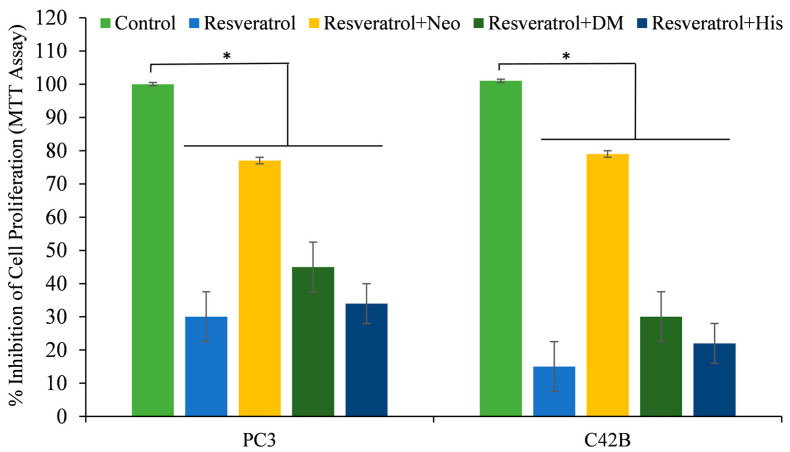
PC3 and C42B prostate cancer cells were exposed to a concentration of 25 µM of resveratrol, either alone or in the presence of the cuprous chelator neocuproine (Neo), the iron chelator desferrioxamine mesylate (DM), or the zinc chelator histidine (His), as shown in the figure. The metal chelators were utilized at a concentration of 50 µM. The MTT assay was conducted 72 h after the treatment. The values presented represent the standard error (±S.E.) of three separate studies. * *p* value < 0.05, compared to control. The cells were also subjected to treatment with each of the three distinct metal-specific chelators individually, serving as negative controls. None of the chelators exhibited a substantial impact on cell proliferation.

In a prior study, the author’s lab discovered that neocuproine, a cuprous chelator that can pass through cell membranes, could inhibit polyphenol-induced oxidative DNA damage, suggesting that nuclear copper plays a role in this process [26,27,38,40]. Figure 4 shows that out of all the cuprous chelators tested, only neocuproine proved to be a substantial shield against resveratrol’s growth inhibitory effects on PC3 and C42B cancer cells. Desferroxamine and histidine, which are iron and zinc chelators, did not have a significant effect, except in PC3 cells where iron and zinc chelators showed some protection against resveratrol growth suppression. Nevertheless, this effect was lower than the inhibition caused by neocuproine.

**Figure 5:**
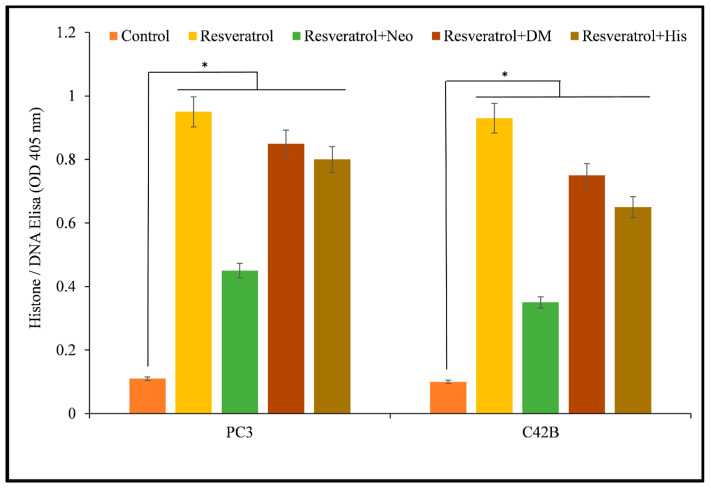
The impact of various metal chelators on the initiation of apoptosis by resveratrol was investigated in two prostate cancer cell lines, PC3 and C42B. The cells were exposed to a concentration of 25 µM of resveratrol, either alone or in combination with the copper chelator neocuproine (Neo), the iron chelator desferrioxamine mesylate (DM), or the zinc chelator histidine (His), as shown in the figure. The metal chelators were utilized at a concentration of 50 µM. A 72 h treatment was conducted and Histone/DNA Elisa assay was performed following the procedures outlined in Section 2. The values presented represent the standard error (±S.E.) of three separate studies. * *p* value < 0.01 when compared to control.

The author also investigated the effects of several metal chelators on resveratrol-induced cell death. This protection was not detected when iron or zinc chelators were present, leading to the conclusion that resveratrol’s anticancer mechanism includes the mobilization of endogenous copper (Figure 5).

### 3.3. Resveratrol Restricts the Growth of Cancer Cells in A Clonogenic Assay

**Figure 6:**
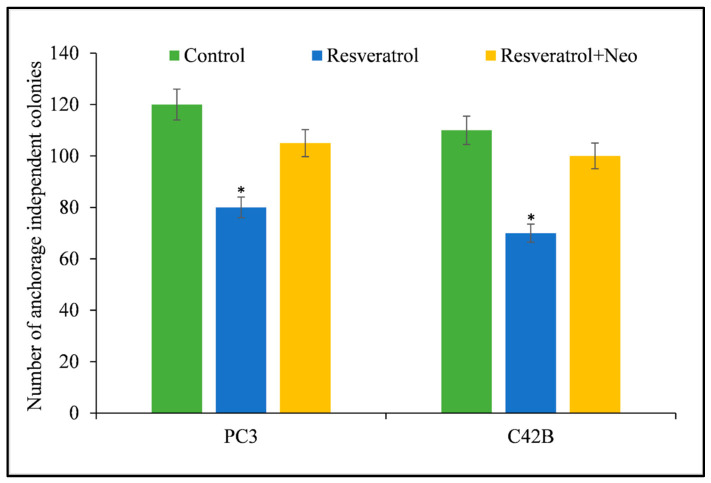
PC3 and C42B prostate cancer cells, (with a count of 3 × 10^4^), were placed in 24-well plates following the procedure outlined in Section 2. The cultures were enhanced with resveratrol at a concentration of 25 µM, either with or without 50 µM of the cuprous chelator neocuproine (Neo). The experiments were conducted three times and the average values are presented. * *p* value < 0.01 when compared to control. Neocuproine was utilized as a negative control in the experiment, and it did not impact the number of cancer cells that were able to grow independently of anchoring.

A clonogenic or colony formation assay is an *in vitro* cell survival experiment that involves the development of a single cell into a colony. Figure 6 demonstrates a decrease in anchorage-independent colonies following the administration of resveratrol to cancer cells. Neocuproine, a cuprous chelator, counteracted the effects of resveratrol providing evidence of the resveratrol’s potential against cancer cell growth.

### 3.4. Resveratrol Induces Cell Death in Cancer Cells through ROS Production

Previous studies have shown that polyphenols can lead to DNA damage in lymphocytes through the production of ROS [26,28,29,32]. A study was conducted to investigate the effects of various ROS scavengers on resveratrol-induced apoptosis in prostate cancer cells. The aim was to validate the findings obtained in cancer cell lines. According to Table 2, the three ROS scavengers had a significant impact on reducing the apoptotic activity induced by resveratrol in the cancer cell lines that were tested. This study provides evidence that ROS plays a role in the pro-oxidant-induced apoptosis caused by resveratrol.

The PC3 and C42B cells were exposed to a 25 µM dose of resveratrol and a variety of ROS scavengers, including TU (700 µM thiourea), SOD (100 µg/mL superoxide dismutase), and CAT (100 µg/mL catalase). Following this, as described in Section 2, histone/DNA ELISA was used to evaluate the effect on apoptosis. The data that have been reported are the mean values with standard error (±S.E.) from a minimum of three separate experiments. In comparison to the untreated control, “apoptosis (folds)” measures the relative increase in cell death.

The effect of scavengers was calculated using the formula {[Resveratrol alone − (Resveratrol + ROS scavenger)]/Resveratrol alone × 100}.

### 3.5. The Antiproliferative Effect of Resveratrol Is Enhanced in Non-Tumorigenic Epithelial Cells When Copper Supplementation Is Administered

**Figure 7:**
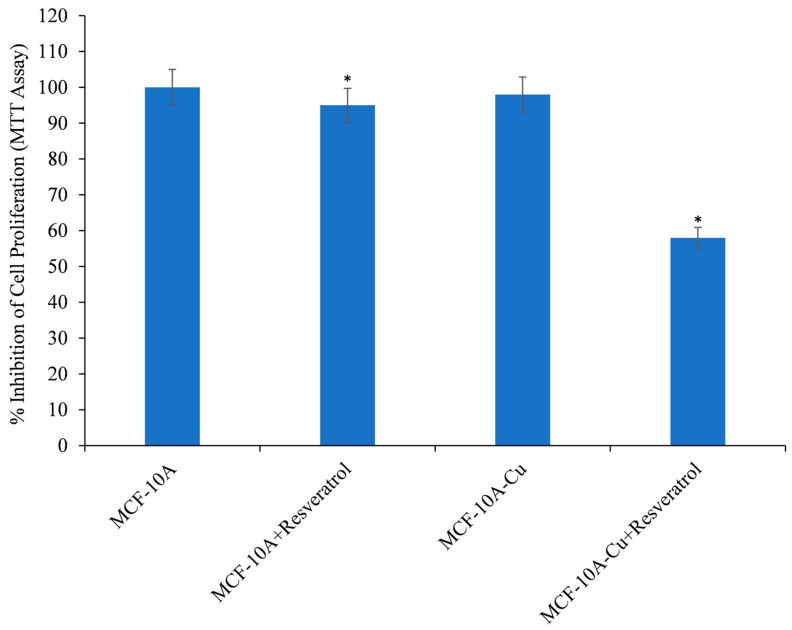
The impact of resveratrol on the suppression of cell proliferation was investigated in MCF-10A (non-tumorigenic epithelial cell line) and MCF-10A cells cultured in a medium supplemented with copper (MCF-10A+Cu). Resveratrol was administered to both “MCF-10A” and “MCF-10A+Cu” cells at a concentration of 25 µM for 72 h. The MCF-10A+Cu cells were cultured on a medium containing 25 µM CuCl_2_. Afterward, cell proliferation was assessed using the MTT assay as described in Section 2. The values presented represent the standard error (±S.E.) of three separate studies. * *p* value < 0.01 when compared to respective control.

The non-tumorigenic epithelial cells, MCF-10A, were cultured in a medium that was enhanced with 25 µM copper. Figure 7 shows that compared to MCF-10A cells without copper supplementation, MCF-10A cells supplemented with copper (MCF-10A+Cu) showed a significant decrease in cell proliferation when exposed to resveratrol. This proves that non-tumorigenic epithelial cells are enhanced to respond to resveratrol-induced cell growth suppression when they are given more copper.

### 3.6. Copper Chelation Reverses the Migration of Cancer Cells That Is Inhibited by Resveratrol

**Figure 8:**
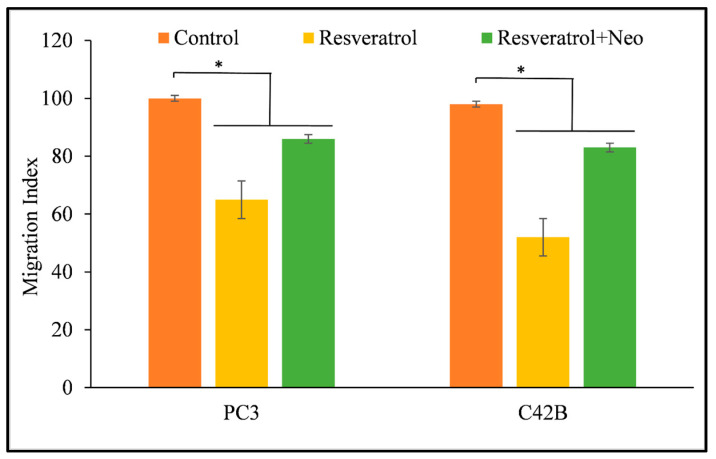
The impact of resveratrol on the migration of PC3 and C42B prostate cancer cells when the copper chelator neocuproine is present. The experiment described in Section 2 involved conducting a cell migration test using 24-well transwell permeable supports with 8 mm pores. The cells were produced in the presence and absence of resveratrol (25 µM) and neocuproine (50 µM). The fluorescence of the moving cells was quantified using an Ultra Multifunctional Microplate Reader. The values presented represent the standard error (±S.E.) of three separate studies. * *p* value < 0.01 when compared to control.

Metastasis is the process by which malignant cells spread to different areas of the body and incorporate themselves into other areas. The capacity of PC3 and C42B prostate cancer cells to migrate was inhibited by resveratrol, which resulted in a reduction in the likelihood that these cells would form metastases. However, the cells were able to regain their capacity to metastasis after being treated with neocuproine, a copper chelator that is membrane-permeable (Figure 8). This provides more evidence that resveratrol-induced malignant cell death involves nuclear copper.

### 3.7. Resveratrol Inhibits the Activity of Copper Transporter CTR1

**Figure 9:**
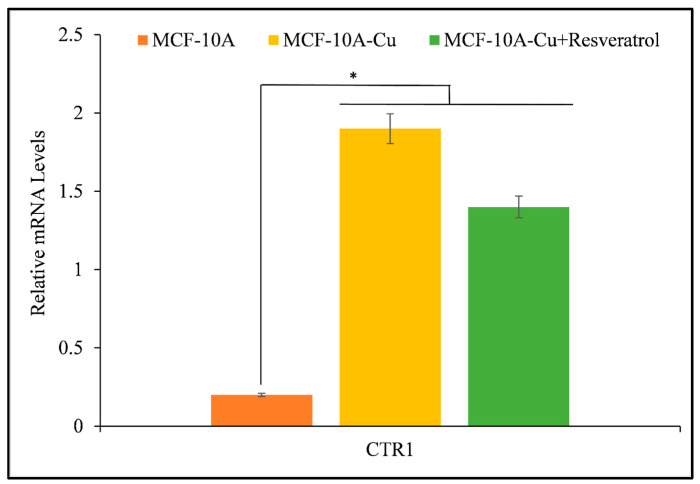
The effect of resveratrol on the suppression of cell proliferation was examined in MCF-10A cells (non-tumorigenic epithelial cells) and MCF-10A cells cultured in a medium supplemented with copper (MCF-10A+Cu). Resveratrol was administered at a concentration of 25 µM to both “MCF-10A” and “MCF-10A+Cu” cells, with the latter being non-tumorigenic cells cultured in a medium containing 25 µM CuCl_2_. The duration of the treatment was 72 h. Afterward, cell proliferation was assessed using the MTT assay as outlined in Section 2. The values presented represent the standard error (±S.E.) of three separate studies. * *p* value < 0.05 when compared to control.

Resveratrol interacts with intracellular copper in both malignant and non-malignant epithelial cells cultured in copper-rich medium, leading to growth inhibition. The author wanted to explore whether copper supplementation could enhance copper transporter expression in non-malignant epithelial cells, considering the higher levels of copper transporter *CTR1* found in malignant cells. Incorporating copper into the growth conditions of MFC-10A cells resulted in a significant increase in the expression of copper transporter *CTR1* (Figure 9) [41,42]. Through the addition of resveratrol to the medium, there was a noticeable decrease in the activity of the copper transporter *CTR1*, indicating that resveratrol contributes to an effect on copper metabolism within cancer cells.

### 3.8. Resveratrol Minimizes Cell Proliferation in MCF-10A Cells Cultivated on Copper-Supplemented Media via Targeted Silencing of CTR1

**Figure 10:**
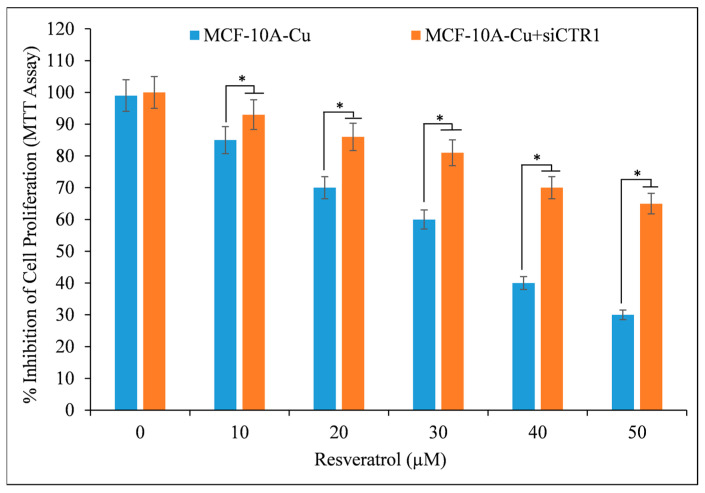
The growth rate of MCF-10A-Cu cells (non-tumorigenic MCF-10A cells grown in a medium containing 25 µM CuCl_2_) was noticeably reduced on exposure to resveratrol after the suppression of *CTR1*. The MCF-10A+Cu cells were initially exposed to resveratrol or specific siRNA against *CTR1* (si*CTR1*) for 48 h, followed by treatment with specified doses of resveratrol after 24 h. The values presented represent the standard error (±S.E.) of three separate studies. * *p* value < 0.05 when compared to control.

The goal was to solidify the understanding that copper is essential in preventing growth stimulated by resveratrol. As a result, siRNA (si*CTR1*) was employed to interfere with the copper transporter *CTR1*. Based on Figure 10, it is evident that the growth suppression caused by resveratrol is more pronounced in MCF-10A cells when *CTR1* is expressed. This can be attributed to the fact that *CTR1* facilitates copper uptake in cells. When copper transporter *CTR1* was inhibited using si*CTR1* in a copper-rich medium, the resveratrol-induced inhibition of MCF-10A cells was observed to be reduced. This study provides conclusive evidence of the interaction between resveratrol and nuclear copper, highlighting the essential role of copper in resveratrol’s ability to effectively inhibit the growth of cancer cells.

## 4. Discussion

This study contributes to the increasing amount of evidence that resveratrol can induce cell death and inhibit proliferation in cancer cells by utilizing a copper-dependent pro-oxidant mechanism. Polyphenols can damage cellular DNA by mobilizing endogenous copper ions and generating ROS [32]. This mechanism had been previously validated via lymphocyte-based investigations that utilized a copper overload [43]. Neocuproine, a copper-specific sequestering agent, significantly reverses the cell growth inhibition caused by resveratrol in cancer cells, while iron and zinc chelators have a negligible effect (Figure 4). Hence, this process of mobilization of endogenous copper ions may be a common mechanism for all plant polyphenols and an important one for the cytotoxic activity of plant polyphenols against cancer cells. It has been demonstrated that polyphenols inhibit the development of cancer cells but have no effect on their nontransformed counterparts. It is worth noting that, in contrast to breast cancer cells, non-tumorigenic epithelial MCF-10A cells have previously been demonstrated to not contain any detectable copper [40,44]. Previous research has shown that cancer cells tend to have higher levels of copper compared to healthy cells. This finding has led to the hypothesis that plant polyphenols may selectively kill cancer cells due to their interaction with copper [32]. Thus, it is logical to assume that the concentrations of resveratrol required would be lower in cancer cells compared to normal cells, owing to the elevated levels of intracellular copper in cancer cells. Multiple studies [45,46,47,48] and this study have demonstrated the cytotoxic effects of lower quantities of resveratrol on cancer cells. To further establish this phenomenon, non-tumorigenic epithelial MCF-10A cells are cultured with copper enrichment to replicate the copper state of cancer cells in the body. This process enhances cellular sensitivity to growth inhibition caused by polyphenols and boosts the expression of the copper transporter *CTR1* (Figure 9). It is worth noting that cancer cells also exhibit an increased expression of copper transporter, which aids in the absorption and storage of excess copper [40,41,42]. As observed, the treatment of MCF-10A cells with copper resulted in elevated levels of *CTR1*-accumulated copper. This caused a higher copper redox state, rendering the cells more susceptible to growth inhibition by resveratrol. In order to further confirm the idea, through the utilization of siRNA to inhibit the expression of the representative copper transporter *CTR1*, the author was able to provide further evidence in favor of the suggested findings. By silencing *CTR1*, it was possible to reverse the resveratrol sensitivity of MCF-10A cells that were grown with copper supplementation (Figure 10). This experiment clearly showed that copper plays a crucial role in the selective cell death induced by resveratrol. As stated earlier, significant levels of copper have been found in numerous types of cancer in humans [33,34]. The reason behind the increased copper concentration in tumors is still unknown. However, research has demonstrated that ceruloplasmin, the primary protein that binds to copper, can potentially stimulate the growth of new blood vessels and is also found in higher levels in cancer cells [33,34,40]. Various pathophysiological mechanisms have been used to explain the correlation between elevated copper levels and the occurrence of hematological cancers. Firstly, there is evidence suggesting that copper plays a role in promoting angiogenesis, a crucial process involved in the growth and metastasis of tumors [40]. In addition to this, endothelial cells exhibit enhanced mobility when exposed to copper ions *in vitro* [40]. In laboratory experiments, copper has been found to enhance the production of fibronectin, a matrix glycoprotein associated with angiogenesis. Also, various studies have shown that a lack of copper can hinder the growth of blood vessels in different types of cancer cells and xenograft systems [40]. In addition, the absence of copper hinders the synthesis of crucial angiogenic cytokines and growth factors, such as IL-1, IL-6, IL-8, tumor necrosis factor, basic fibroblast growth factor, and vascular endothelial growth factor [40]. Based on these findings, it has been discovered that copper plays a crucial role in angiogenesis. Therefore, it has also been suggested that reducing copper levels using copper chelators could potentially have beneficial effects in preventing and treating cancer [17]. Several clinical trials have also examined the effectiveness of copper chelators in inhibiting angiogenesis [33,34]. It is widely recognized that copper plays a significant role beyond just promoting angiogenesis. It is essential for intracellular signaling and the spread of tumors in cancer. Cell migration and invasion from the initial tumor location require the degradation of cell-to-cell connections in the target tissue [40]. Cell invasion relies on the epithelial–mesenchymal transition, disrupting cell-to-cell junctions due to the downregulation of E-cadherin, a protein responsible for cell-to-cell adhesion. Usually, a sophisticated network involving the Snail repressor and the copper-dependent LOX-like proteins controls the transcription of E-cadherin. It has been observed that copper can influence the regulation of the epithelial–mesenchymal transition [40,49,50]. After discussing the crucial role of copper in cancer growth, it is important to emphasize that the elevated copper levels present in cancer can be specifically targeted to eliminate cancer cells. The research presented here explores the valuable application of cancer cells’ higher copper levels in harnessing the chemopreventive or potential therapeutic effects of resveratrol or plant-derived polyphenols. It can be said that plant polyphenols exhibit a shared mechanism for their ability to kill cancer cells, which involves the movement of nuclear copper ions. Research findings suggest that a variety of polyphenols and Cu(II) exhibit anticancer properties by causing oxidative DNA damage through ROS production and intercalation of the complex into DNA [26,27,28,29,30,38,51,52,53]. It has been demonstrated that the polyphenol-Cu(II) system can break down the cellular DNA in an oxidative manner. This DNA breakage results in the production of ROS and the temporary conversion of Cu(II) to Cu(I). The ROS probably plays a role in the DNA damage and cell death induced by polyphenols. Thus, the proposed mechanism suggests an alternative way in which polyphenols, capable of mobilizing and reducing endogenous copper, exert their inhibiting effects. This pathway relies on copper and does not require enzymes. As a result, there is no involvement of Fas and mitochondria-mediated programmed cell death [40]. Several studies have demonstrated that certain drugs used to treat cancer can trigger cell death through mechanisms that do not rely on caspases or mitochondria [54,55]. Additionally, this process is associated with an increase in levels of ROS within cells [56,57,58]. It aligns with the theory that plant polyphenols might play a part in the generation of ROS by aiding the redox cycling of copper bound to chromatin. Published articles have already reported that the majority of agents used to evade or kill cancer cells, such as ionizing radiation, chemotherapeutic agents, and targeted therapies, work by generating ROS that disrupt key steps in the cell cycle [59,60]. Polyphenols primarily generate ROS, but they also have secondary effects on various transcription factors (NF-kB, P53, Myc, Nrf2, etc.) and pro- and anti-apoptotic proteins (Bcl-2, Bax, etc.) [40,60].

## 5. Conclusions

In light of the research reported here, it is safe to draw the conclusion that resveratrol and other plant polyphenols possess anticancer and chemopreventive effects which happen through the mobilization of nuclear copper and subsequent pro-oxidant action. The fact that polyphenols with different chemical structures have different chemopreventive effects and selective cytotoxicity towards cancer cells can be better understood by considering this common shared mechanism. Although plant polyphenols may have anticancer therapeutic effects, the present work is important for laying the groundwork for the synthesis of new, improved anticancer medications and formulations that have a longer systemic half-life, higher bioavailability, and more effective lead compounds.

## Figures and Tables

**Table 1:** Primer sets.

Gene Name	Forward Primer	Reverse Primer
*CTR1*	5′- GCT GGA AGA AGG CAG TGG TA-3′	5′- AAA GAG GAG CAA GAA GGG ATG -3′
*GADPH*	5′- TGG GTG TGA ACC ATG AGA AGT -3′	5′- TGA GTC CTT CCA CGA TAC CAA -3′

**Table 2:** Effect of ROS scavengers on resveratrol activity in prostate cancer cells.

Cell Line	Treatment	Apoptosis (Folds)	Inhibition of Apoptosis (%)
PC3	Untreated	-	-
	Resveratrol	2.32 ± 0.2	-
	+TU	1.11 ± 0.2	52.15
	+SOD	1.27 ± 0.1	45.25
	+CAT	1.38 ± 0.1	40.15
C42B	Untreated	-	-
	Resveratrol	2.41 ± 0.3	-
	+TU	1.05 ± 0.1	45.22
	+SOD	1.18 ± 0.2	51.03
	+CAT	1.23 ± 0.1	48.96

## Data Availability

The original contributions presented in the study are included in the article, further inquiries can be directed to the author.

## References

[1] Farhan M., Rizvi A. (2023). The Pharmacological Properties of Red Grape Polyphenol Resveratrol: Clinical Trials and Obstacles in Drug Development. Nutrients.

[2] Renaud S., de Lorgeril M. (1992). Wine, alcohol, platelets, and the French paradox for coronary heart disease. Lancet.

[3] Fragopoulou E., Antonopoulou S. (2020). The French paradox three decades later: Role of inflammation and thrombosis. Clin. Chim. Acta.

[4] De Sá Coutinho D., Pacheco M.T., Frozza R.L., Bernardi A. (2018). Anti-Inflammatory Effects of Resveratrol: Mechanistic Insights. Int. J. Mol. Sci..

[5] Michno A., Grużewska K., Ronowska A., Gul-Hinc S., Zyśk M., Jankowska-Kulawy A. (2022). Resveratrol Inhibits Metabolism and Affects Blood Platelet Function in Type 2 Diabetes. Nutrients.

[6] Colica C., Milanović M., Milić N., Aiello V., De Lorenzo A., Abenavoli L. (2018). A Systematic Review on Natural Antioxidant Properties of Resveratrol. Nat. Prod. Commun..

[7] Duta-Bratu C.-G., Nitulescu G.M., Mihai D.P., Olaru O.T. (2023). Resveratrol and Other Natural Oligomeric Stilbenoid Compounds and Their Therapeutic Applications. Plants.

[8] Pannu N., Bhatnagar A. (2019). Resveratrol: From enhanced biosynthesis and bioavailability to multitargeting chronic diseases. Biomed. Pharm..

[9] Andrade S., Ramalho M.J., Pereira M.D.C., Loureiro J.A. (2018). Resveratrol Brain Delivery for Neurological Disorders Prevention and Treatment. Front. Pharm..

[10] Xiao Q., Zhu W., Feng W., Lee S.S., Leung A.W., Shen J., Gao L., Xu C. (2019). A Review of Resveratrol as a Potent Chemoprotective and Synergistic Agent in Cancer Chemotherapy. Front. Pharmacol..

[11] Frankel E.N., Waterhouse A.L., Kinsella J.E. (1993). Inhibition of Human LDL Oxidation by Resveratrol. Lancet.

[12] Belguendouz L., Fremont L., Linard A. (1997). Resveratrol inhibits metal ion-dependent and independent peroxidation of porcine low-density lipoproteins. Biochem. Pharmacol..

[13] Ali M., Benfante V., Di Raimondo D., Salvaggio G., Tuttolomondo A., Comelli A. (2024). Recent Developments in Nanoparticle Formulations for Resveratrol Encapsulation as an Anticancer Agent. Pharmaceuticals.

[14] Varoni E.M., Lo Faro A.F., Sharifi-Rad J., Iriti M. (2016). Anticancer molecular mechanisms of resveratrol. Front. Nutr..

[15] Zulueta A., Caretti A., Signorelli P., Ghidoni R. (2015). Resveratrol: A potential challenger against gastric cancer. World J. Gastroenterol..

[16] Aluyen J.K., Ton Q.N., Tran T., Yang A.E., Gottlieb H.B., Bellanger R.A. (2012). Resveratrol: Potential as anticancer agent. J. Diet. Suppl..

[17] Colin D., Limagne E., Jeanningros S., Jacquel A., Lizard G., Athias A., Gambert P., Hichami A., Latruffe N., Solary E. (2011). Endocytosis of resveratrol via lipid rafts and activation of downstream signaling pathways in cancer cells. Cancer Prev. Res..

[18] Fulda S., Debatin K.M. (2006). Resveratrol modulation of signal transduction in apoptosis and cell survival: A mini-review. Cancer Detect. Prev..

[19] Lin H.Y., Tang H.Y., Davis F.B., Davis P.J. (2011). Resveratrol and apoptosis. Ann. N. Y. Acad. Sci..

[20] Whitlock N.C., Baek S.J. (2012). The anticancer effects of resveratrol: Modulation of transcription factors. Nutr. Cancer.

[21] Paller C.J., Rudek M.A., Zhou X.C., Wagner W.D., Hudson T.S., Anders N., Hammers H.J., Dowling D., King S., Antonarakis E.S. (2015). A phase i study of muscadine grape skin extract in men with biochemically recurrent prostate cancer: Safety, tolerability, and dose determination. Prostate.

[22] Kjaer T.N., Ornstrup M.J., Poulsen M.M., Jorgensen J.O., Hougaard D.M., Cohen A.S., Neghabat S., Richelsen B., Pedersen S.B. (2015). Resveratrol reduces the levels of circulating androgen precursors but has no effect on, testosterone, dihydrotestosterone, psa levels or prostate volume. A 4-month randomised trial in middle-aged men. Prostate.

[23] Howells L.M., Berry D.P., Elliott P.J., Jacobson E.W., Hoffmann E., Hegarty B., Brown K., Steward W.P., Gescher A.J. (2011). Phase i randomized, double-blind pilot study of micronized resveratrol (srt501) in patients with hepatic metastases—Safety, pharmacokinetics, and pharmacodynamics. Cancer Prev. Res..

[24] Patel K.R., Brown V.A., Jones D.J., Britton R.G., Hemingway D., Miller A.S., West K.P., Booth T.D., Perloff M., Crowell J.A. (2010). Clinical pharmacology of resveratrol and its metabolites in colorectal cancer patients. Cancer Res..

[25] Zhu W., Qin W., Zhang K., Rottinghaus G.E., Chen Y.C., Kliethermes B., Sauter E.R. (2012). Trans-resveratrol alters mammary promoter hypermethylation in women at increased risk for breast cancer. Nutr. Cancer.

[26] Alhasawi M.A.I., Aatif M., Muteeb G., Alam M.W., Oirdi M.E., Farhan M. (2022). Curcumin and Its Derivatives Induce Apoptosis in Human Cancer Cells by Mobilizing and Redox Cycling Genomic Copper Ions. Molecules.

[27] Farhan M., El Oirdi M., Aatif M., Nahvi I., Muteeb G., Alam M.W. (2023). Soy Isoflavones Induce Cell Death by Copper-Mediated Mechanism: Understanding Its Anticancer Properties. Molecules.

[28] Arif H., Sohail A., Farhan M., Rehman A.A., Ahmad A., Hadi S.M. (2018). Flavonoids-induced redox cycling of copper ions leads to generation of reactive oxygen species: A potential role in cancer chemoprevention. Int. J. Biol. Macromol..

[29] Farhan M., Zafar A., Chibber S., Khan H.Y., Arif H., Hadi S.M. (2015). Mobilization of copper ions in human peripheral lymphocytes by catechins leading to oxidative DNA breakage: A structure activity study. Arch. Biochem. Biophys..

[30] Farhan M., Aatif M., Hadi S.M., Ahmad A., Chakraborti S., Ray B.K., Roychoudhury S. (2022). Mechanism of Gallic Acid Anticancer Activity Through Copper-Mediated Cell Death. Handbook of Oxidative Stress in Cancer: Mechanistic Aspects.

[31] Fukuhara K., Miyata N. (1998). Resveratrol as a new type of DNA-cleaving agent. Bioorganic Med. Chem. Lett..

[32] Hadi S.M., Bhat S.H., Azmi A.S., Hanif S., Shamim U., Ullah M.F. (2007). Oxidative breakage of cellular DNA by plant polyphenols: A putative mechanism for anticancer properties. Semin. Cancer Biol..

[33] Farhan M., Rizvi A. (2022). Understanding the prooxidant action of plant polyphenols in the cellular microenvironment of malignant cells: Role of copper and therapeutic implications. Front. Pharmacol..

[34] Gupte A., Mumper R.J. (2009). Elevated copper and oxidative stress in cancer cells as a target for cancer treatment. Cancer Treat. Rev..

[35] Rizvi A., Furkan M., Naseem I. (2017). Physiological serum copper concentrations found in malignancies cause unfolding induced aggregation of human serum albumin *in vitro*. Arch. Biochem. Biophys..

[36] Rudzińska A., Juchaniuk P., Oberda J., Wiśniewska J., Wojdan W., Szklener K., Mańdziuk S. (2023). Phytochemicals in Cancer Treatment and Cancer Prevention—Review on Epidemiological Data and Clinical Trials. Nutrients.

[37] Jomová K., Hudecova L., Lauro P., Simunkova M., Alwasel S.H., Alhazza I.M., Valko M. (2019). A Switch between Antioxidant and Prooxidant Properties of the Phenolic Compounds Myricetin, Morin, 3′,4′-Dihydroxyflavone, Taxifolin and 4-Hydroxy-Coumarin in the Presence of Copper(II) Ions: A Spectroscopic, Absorption Titration and DNA Damage Study. Molecules.

[38] Farhan M. (2022). Naringin’s Prooxidant Effect on Tumor Cells: Copper’s Role and Therapeutic Implications. Pharmaceuticals.

[39] Tice R.R., Agurell E., Anderson D., Burlinson B., Hartmann A., Kobayashi H., Miyamae Y., Rojas E., Ryu J.C., Sasaki Y.F. (2000). Single cell gel/comet assay: Guidelines for *in vitro* and *in vivo* genetic toxicology testing. Environ. Mol. Mutagen..

[40] Khan H.Y., Zubair H., Faisal M., Ullah M.F., Farhan M., Sarkar F.H., Ahmad A., Hadi S.M. (2014). Plant polyphenol induced cell death in human cancer cells involves mobilization of intracellular copper ions and reactive oxygen species generation: A mechanism for cancer chemopreventive action. Mol. Nutr. Food Res..

[41] Ge E.J., Bush A.I., Casini A., Cobine P.A., Cross J.R., DeNicola G.M., Dou Q.P., Franz K.J., Gohil V.M., Gupta S. (2022). Connecting copper and cancer: From transition metal signalling to metalloplasia. Nat. Rev. Cancer.

[42] Wang X., Zhou M., Liu Y., Si Z. (2023). Cope with Copper: From Copper Linked Mechanisms to Copper-Based Clinical Cancer Therapies. Cancer Lett..

[43] Khan H.Y., Zubair H., Ullah M.F., Ahmad A., Hadi S.M. (2011). Oral administration of copper to rats leads to increased lymphocyte cellular DNA degradation by dietary polyphenols: Implications for a cancer preventive mechanism. Biometals.

[44] Daniel K.G., Chen D., Orlu S., Cui Q.C., Miller F.R., Dou Q.P. (2005). Clioquinol and pyrrolidine dithiocarbamate complex with copper to form proteasome inhibitors and apoptosis inducers in human breast cancer cells. Breast Cancer Res..

[45] Miłek M., Marcinčáková D., Legáth J. (2019). Polyphenols Content, Antioxidant Activity, and Cytotoxicity Assessment of *Taraxacum officinale* Extracts Prepared through the Micelle-Mediated Extraction Method. Molecules.

[46] Kanner J. (2023). Food Polyphenols as Preventive Medicine. Antioxidants.

[47] Salehi B., Mishra A.P., Nigam M., Sener B., Kilic M., Sharifi-Rad M., Fokou P.V.T., Martins N., Sharifi-Rad J. (2018). Resveratrol: A Double-Edged Sword in Health Benefits. Biomedicines.

[48] Moreira-Pinto B., Costa L., Felgueira E., Fonseca B.M., Rebelo I. (2021). Low Doses of Resveratrol Protect Human Granulosa Cells from Induced-Oxidative Stress. Antioxidants.

[49] Wang Z., Jin D., Zhou S., Dong N., Ji Y., An P., Wang J., Luo Y., Luo J. (2023). Regulatory roles of copper metabolism and cuproptosis in human cancers. Front. Oncol..

[50] Bian C., Zheng Z., Su J., Chang S., Yu H., Bao J., Xin Y., Jiang X. (2023). Copper homeostasis and cuproptosis in tumor pathogenesis and therapeutic strategies. Front. Pharmacol..

[51] Duda-Chodak A., Tarko T. (2023). Possible Side Effects of Polyphenols and Their Interactions with Medicines. Molecules.

[52] Farhan M., Khan H.Y., Oves M., Al-Harrasi A., Rehmani N., Arif H., Hadi S.M., Ahmad A. (2016). Cancer Therapy by Catechins Involves Redox Cycling of Copper Ions and Generation of Reactive Oxygen Species. Toxins.

[53] Arif H., Rehmani N., Farhan M., Ahmad A., Hadi S.M. (2015). Mobilization of Copper ions by Flavonoids in Human Peripheral Lymphocytes Leads to Oxidative DNA Breakage: A Structure Activity Study. Int. J. Mol. Sci..

[54] Bhadra K. (2022). A Mini Review on Molecules Inducing Caspase-Independent Cell Death: A New Route to Cancer Therapy. Molecules.

[55] Leist M., Jäättelä M. (2001). Four deaths and a funeral: From caspases to alternative mechanisms. Nat. Rev. Mol. Cell Biol..

[56] Mu Q., Najafi M. (2021). Resveratrol for targeting the tumor microenvironment and its interactions with cancer cells. Int. Immunopharmacol..

[57] Bian Y., Wei J., Zhao C., Li G. (2020). Natural Polyphenols Targeting Senescence: A Novel Prevention and Therapy Strategy for Cancer. Int. J. Mol. Sci..

[58] Varesi A., Chirumbolo S., Campagnoli L.I.M., Pierella E., Piccini G.B., Carrara A., Ricevuti G., Scassellati C., Bonvicini C., Pascale A. (2022). The Role of Antioxidants in the Interplay between Oxidative Stress and Senescence. Antioxidants.

[59] Watson J. (2013). Oxidants, Antioxidants and the Current Incurability of Metastatic Cancers. Open Biol..

[60] Zucchi A., Claps F., Pastore A.L., Perotti A., Biagini A., Sallicandro L., Gentile R., Caglioti C., Palazzetti F., Fioretti B. (2023). Focus on the Use of Resveratrol in Bladder Cancer. Int. J. Mol. Sci..

